# The Rubber Contest

**Published:** 1874-07

**Authors:** 


### Editorial.

### THE RUBBER CONTEST.

It is with pleasure that we give place to the following statement from the Ohio State Dental Society's Committee on patents. It contains a clear and unmistakable indication as to the course the dentists should pursue in reference to the Rubber question, for the present at least.

We have so often said to the profession: Stand still till a final discussion of this contest is had, and not furnish the means to the Vulcanite Company, by which the fight shall be sustained, that it seems unnecessary to say anything more; but with some, line upon line is needed. And now, again we say, pay not one cent to that Company, till they *prove their title clear*; nor even receive a license gratuitously, but what any have to contribute, appropriate it to the defense. If the profession had refused to give support to this abominable iniquity it would have been dead long ago. ED.

### TO THE DENTAL PROFESSION.

The fact has become well known to the Dentists of Ohio that Judge Shepley has decided the case of the Goodyear Dental Vulcanite Co., *et. al.* vs Daniel H. Smith in favor of the complainants, and that, in the decision, he sustains the validity of the Cumming's patent. To any one at all conversant with patent law, the decision presents nothing at all discouraging.

It may perplex, but should not dishearten those, who have from the first believed that the Cumming's patent is a fraud upon the great body of Dentists in the United States, who, for years before the issuing of letters patent to Cummings, had, with the consent of the patentee Goodyear, or his assigns, used the material and without any assistance, or intelligible suggestion from Cummings, perfectly understood and used the process, the monopoly of which is now claimed under said patent. Certainly no such assistance, or suggestion was furnished by the caveat, or the two successive applications for a patent filed by Cummings, or the specifications of the original patent granted him as late as 1864, and the dentists acquired the right to use this process, either by the exercise of their own inventive genius, or by the aid of those who were anxious to extend the uses to which hard rubber, or vulcanite might be applied, without the aid of Cummings and long before he himself really understood what the process was.

The decision considered as an attempt to supersede logic by sophistry and to over-ride established law by special pleading may be considered a success, but as an attempt to announce the law applicable to the case and fairly deducible from the issues made and the facts proven, it is a failure, and the cases now pending in Ohio, or which may hereafter be brought are in no manner affected by it.

This case, as well as all others which have yet been tried, involving the validity of the Cumming's patent, has been tried at the East and under every possible disadvantage. A case is now prepared and ready for trial at Detroit, Michigan, which it is supposed will be tried at farthest by next October. This case presents all that was in the Smith case and some very important points for the defence in addition and will be heard by judges who are unprejudiced and strictly impartial.

The justice of the defense and the iniquity of compelling the dentists to pay tribute to those who claim under a patent, so utterly without a substantial foundation in law or equity to rest upon, seem so clear, that but one result ought to follow a fair trial. No dentist should feel at all disheartened, or, by taking a license from the company, contribute means to strengthen the cause of those who seek to maintain this unjust monopoly and thus, at the same time, weaken the defense. Let the motto of every dentist be, "Millions, if need be, for defense, but not one cent for tribute."

The attorneys for the Dentists of Ohio feel entirely confident that no injunction will be allowed, until after the trial of the Detroit case, which will no doubt be decisive of all cases pending in this Circuit and their confidence in that case is such, that they desire there should be no break in the ranks. If the case in Detroit should be decided as the Smith case has been, it will certainly be taken to the Supreme Court of the United States, and we trust no dentist will do homage, or pay tribute to Bacon until he is finally compelled to do so. We expect in the end to rejoice with you over the defeat of this iniquity and to present to you our *dear* Bacon, well cooked.

F. H. Rehwinkle, Chairman of Committee.

H. A. Smith, Secretary.

Cincinnati, June 25, 1874.

